# Supplementary material for: Deep Imputation for Skeleton data (DISK) for behavioral science
Source: Nat Methods. 2025 Dec 4;23(1):236–47. doi: 10.1038/s41592-025-02893-y (PMC12791013; doi:10.1038/s41592-025-02893-y)
Supplement: Supplementary file 1 — Supplementary Figs. 1–5 and Tables 1 and 2. [file 41592_2025_2893_MOESM1_ESM.pdf]

nature methods

Supplementary information https://doi.org/10.1038/s41592-025-02893-y

Deep Imputation for Skeleton data (DISK) for behavioral science

In the format provided by the authors and unedited

Supplementary Figures and Tables

Architecture	Hyperparameters	#parameters FL2	#parameters DF3D
DISK (transformer)	4 layers x 128 dim x 8 heads	408,579	412,419
DISK-proba	4 layers x 128 dim x 8 heads	408,966	412,806
GRU	3 layers x 512 units	11,139,096	11,553,906
GRU-proba	3 layers x 512 units	11,163,720	11,670,870
TCN	4 layers x 256 units, kernel size=3	1,045,560	1,298,310
TCN	4 layers x 512 units, kernel size=3	4,055,352	4,523,142
ST-GCN	4 layers, 64 hidden size, kernel size=3	4,491,148	4,503,808
ST-GCN	4 layers, 128 hidden size, kernel size=3	17,937,452	17,950,112
ST-GCN	4 layers, 256 hidden size, kernel size=3	71,697,772	71,710,432
STS-GCN	4 layers x 256 units, kernel size=3	135,582	517,182
STS-GCN	4 layers x 512 units, kernel size=3	140,702	522,302

Table 1: **Number of parameters for each network** used for FL2 (8 keypoints) and DF3D (38 keypoints) for an input sequence of length 60.

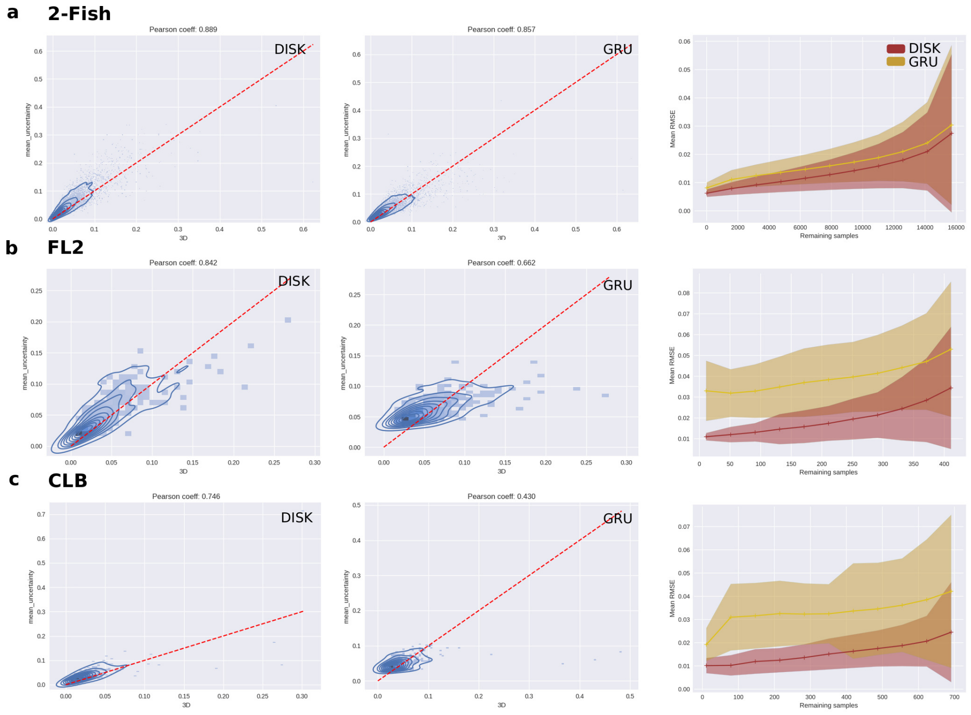

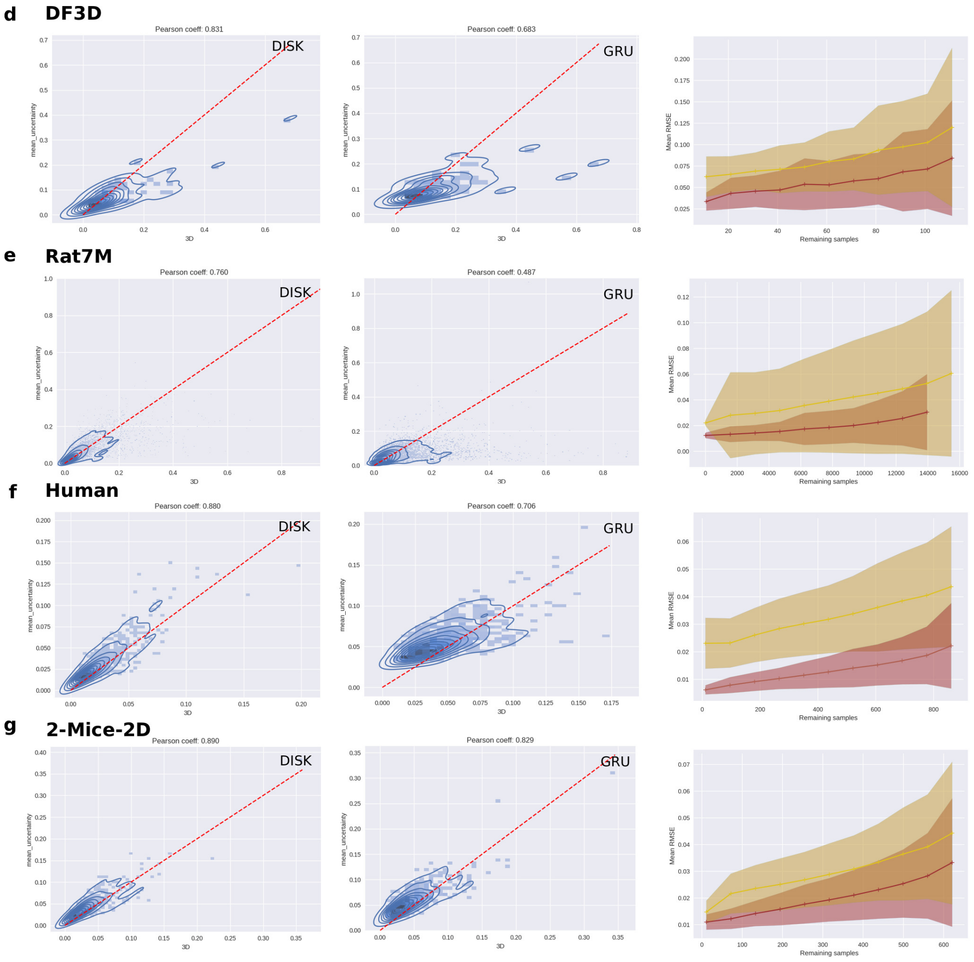

Fig. 1: **Estimated error correlation plots with Pearson correlation coefficient (first and second column) and RMSE after filtering based on estimated error plots (third column) for DISK-proba and GRU-proba for all tested datasets**, The red line shows y = x. For the third column, data are presented as mean values +/- standard deviation.

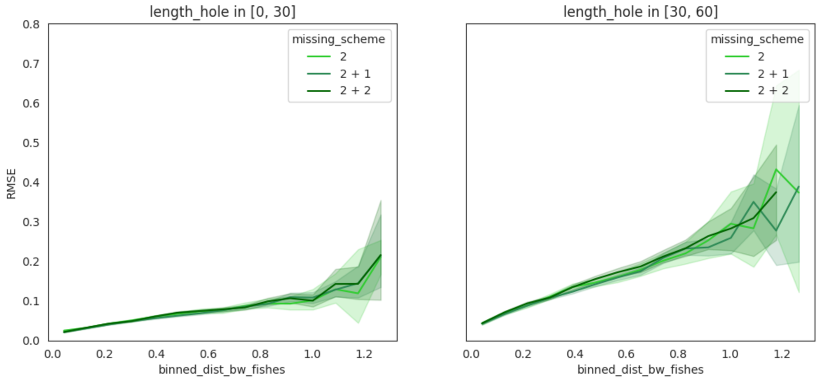

Fig. 2: **RMSE with respect to the distance between the two fish and the number and scheme of missing keypoints for short gaps (upper panel – up to 30 frames) and long gaps (lower panel – from 30 to 60 frames) for the cases 2, 2 + 1, 2 + 2.** These are the missing cases left out from Fig. 4. Results obtained with a DISK model trained with uniform probability. Data are presented as mean values +/- 95% confidence interval.

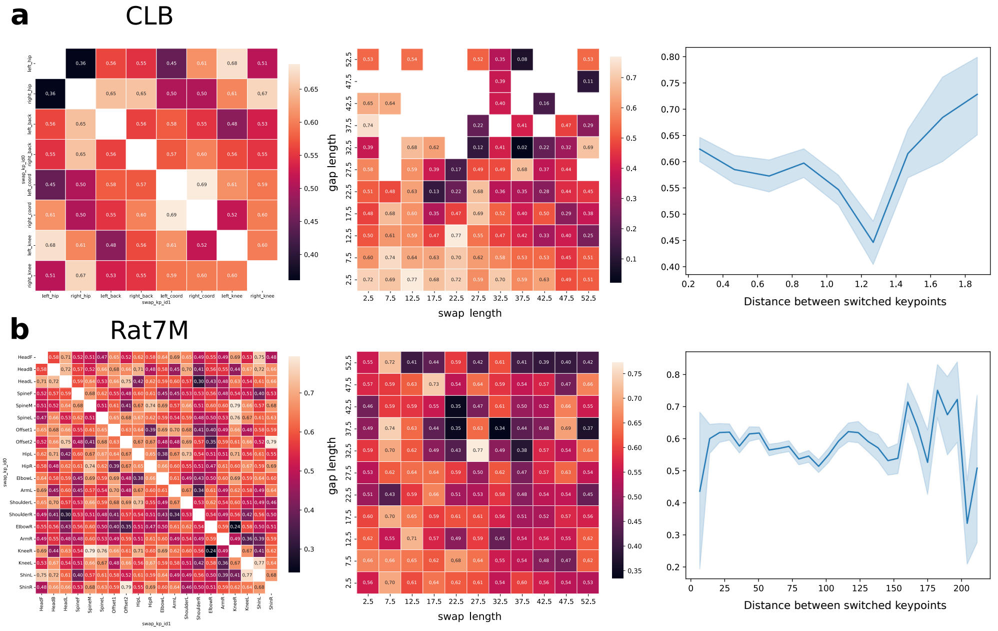

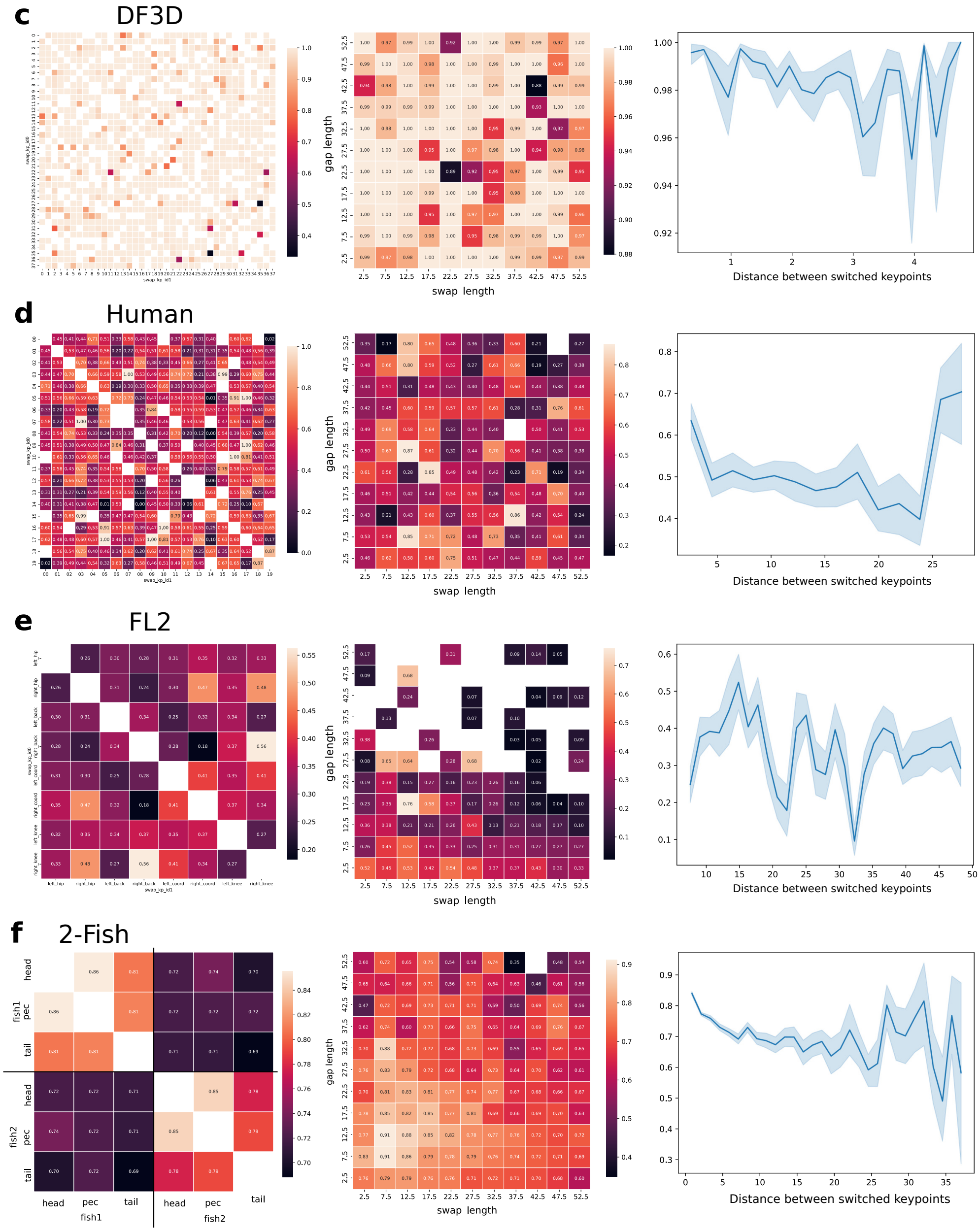

Fig. 3: **PCK@0.01 values for *switch* experiments** Similar plots as Extended Data Fig. 6 d - f for the other datasets. As the pairs of keypoints being switched are chosen randomly, blank matrix squares correspond to combinations not drawn in the limited test set. Gap lengths are expressed in frames. For the third column, data are presented as mean values +/- standard deviation.

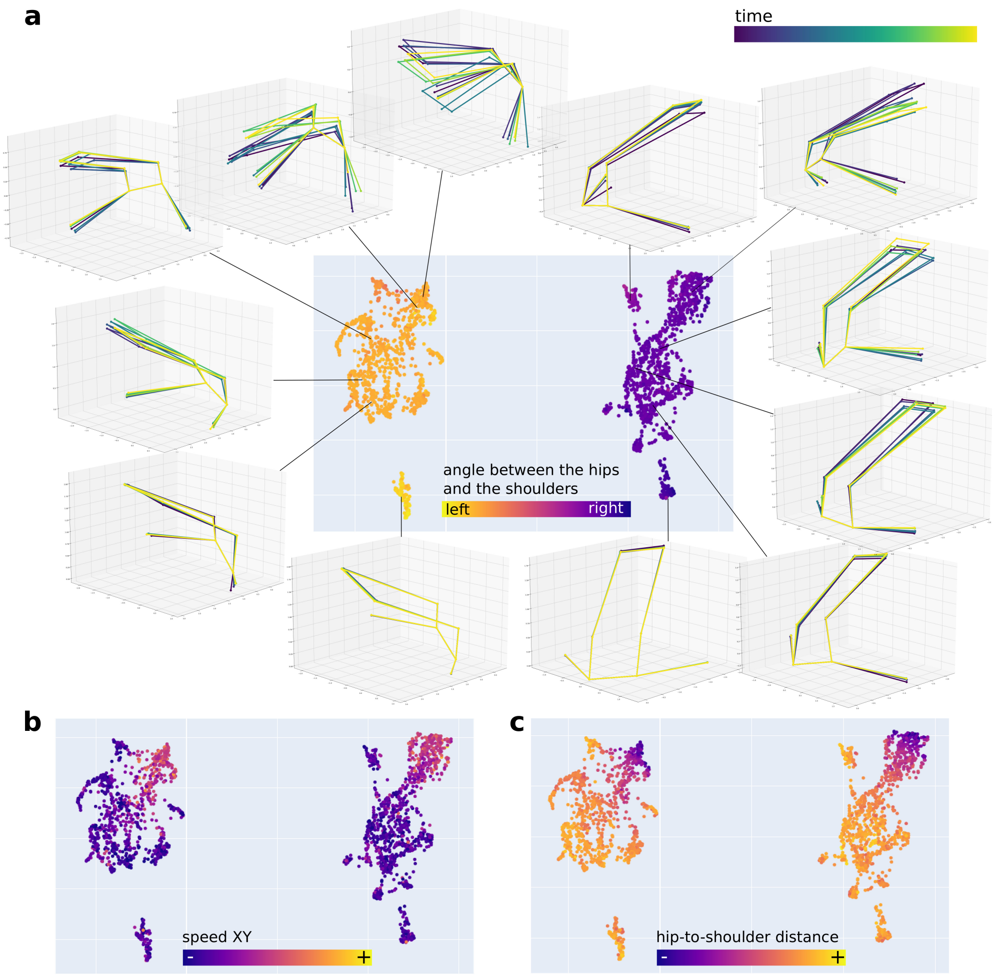

Fig. 4: **DISK learns meaningful representations of 1 second-long sequences of the Mouse FL2 dataset. a - c** Projection of DISK latent space of the sequences via U-map colored by **a** the angle between the hips and the shoulders – reflecting the global direction of the body, **b** the speed in the x-y plane, and **c** the hip-to-shoulder distance – which varies depending on the posture and locomotion behavior. One point on the U-map corresponds to one sequence. **a** 3D skeleton representations of randomly selected sequences.

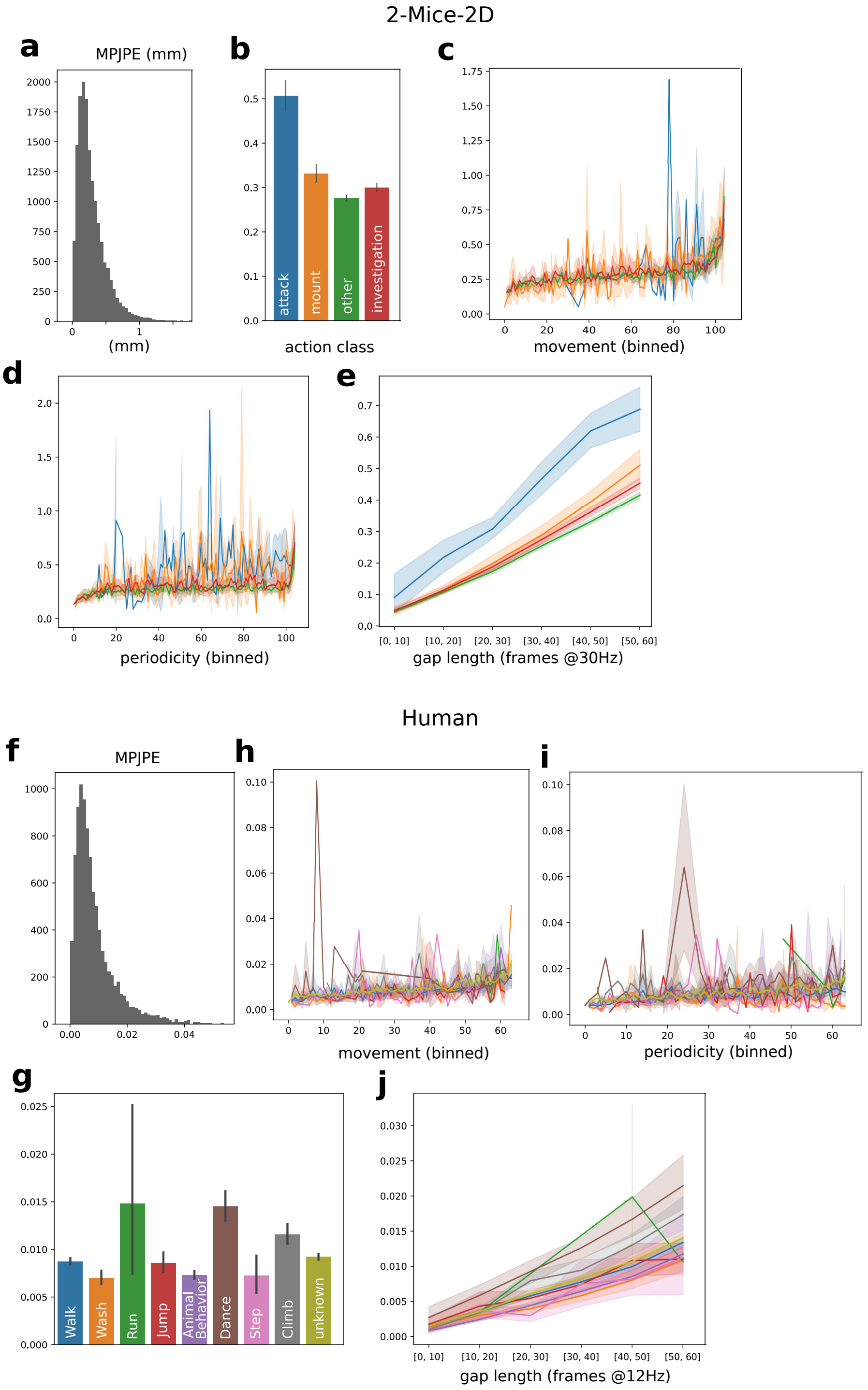

Fig. 5: **Analysis of DISK performance with respect to action classes, quantity of movement, periodicity, and gap length**, on **a - e** 2-Mice-2D and **f - j**

Fig. 5: Human datasets. **a & f** Histogram of averaged MPJPE per sample. **b & g** Averaged MPJPE per sample separated by action class. **c & h** Averaged MPJPE per sample according to the average overall movement present in the sample. **d & i** Averaged MPJPE per sample according to periodicity. **e & j** Averaged MPJPE per sample according to gap length (expressed in frames). For the third column, data are presented as mean values +/- 95% confidence interval for both bar and line charts.

Method	RMSE on fish1	RMSE on fish2
linear interpolation	0.0826	0.0806
GRU 2-fish	0.0297	0.0319
GRU 1-fish	0.0315	0.0345
GRU-proba 2-fish	0.0304	0.0323
GRU-proba 1-fish	0.0323	0.0342
DISK 2-fish	**0.0274**	**0.0295**
DISK 1-fish	0.0297	0.0336
DISK-proba 2-fish	**0.0273**	**0.0294**
DISK-proba 1-fish	0.0294	0.0326

Table 2: **Comparison between the models tested on the 2-fish dataset** (all keypoints taken together) **and the models tested separately on 1 fish or the other** (one model trained on keypoints from *first* fish only, and one model trained on keypoints from the *second* fish only). Mean RMSE of 5 test runs is reported. Best models are indicated in bold.
